# Genomic correlation: harnessing the benefit of combining two unrelated populations for genomic selection

**DOI:** 10.1186/s12711-015-0162-0

**Published:** 2015-11-02

**Authors:** Laercio R. Porto-Neto, William Barendse, John M. Henshall, Sean M. McWilliam, Sigrid A. Lehnert, Antonio Reverter

**Affiliations:** CSIRO Agriculture, Queensland Bioscience Precinct, St. Lucia, QLD 4067 Australia

## Abstract

**Background:**

The success of genomic selection in animal breeding hinges on the availability of a large reference population on which genomic-based predictions of additive genetic or breeding values are built. Here, we explore the benefit of combining two unrelated populations into a single reference population.

**Methods:**

The datasets consisted of 1829 Brahman and 1973 Tropical Composite cattle with measurements on five phenotypes relevant to tropical adaptation and genotypes for 71,726 genome-wide single nucleotide polymorphisms (SNPs). The underlying genomic correlation for the same phenotype across the two breeds was explored on the basis of consistent linkage disequilibrium (LD) phase and marker effects in both breeds.

**Results:**

The proportion of genetic variance explained by the entire set of SNPs ranged from 37.5 to 57.6 %. Estimated genomic correlations were drastically affected by the process used to select SNPs and went from near 0 to more than 0.80 for most traits when using the set of SNPs with significant effects and the same LD phase in the two breeds. We found that, by carefully selecting the subset of SNPs, the missing heritability can be largely recovered and accuracies in genomic predictions can be improved six-fold. However, the increases in accuracy might come at the expense of large biases.

**Conclusions:**

Our results offer hope for the effective implementation of genomic selection schemes in situations where the number of breeds is large, the sample size within any single breed is small and the breeding objective includes many phenotypes.

## Background

To improve the accuracy of genomic selection, large reference populations are usually recommended for estimating genome-based predictions of additive genetic effects or breeding values [1–3]. When large reference populations are not available for a particular breed, it has been proposed that the combined data from different breeds be used to generate genomic breeding values in order to increase the effective size of the reference population [4–6]. Using simulated data, De Roos et al. [7] showed that a multi-breed reference population could improve the accuracy of genome-based estimated breeding values (GEBV), provided that markers were sufficiently dense and the divergence between breeds was not too great. However, combining data from many breeds into a reference population has not always improved the prediction accuracy of breeding values in the validation sample [8–12] and has led to lower prediction accuracies than would have been expected based on the increase in the size of the training population [13]. While prediction accuracy is usually improved for the breed with less data, prediction accuracy for the breed with more data is usually not improved [14].

To improve prediction accuracy through the analysis of combined data, there must be a positive genetic correlation for the phenotype of interest between the two breeds. In addition, since genomic prediction that uses genome-wide single nucleotide polymorphisms (SNPs) is almost entirely based on linkage disequilibrium (LD) between the SNP alleles and the causative allele(s) at the quantitative trait locus (QTL), combining data with inconsistent phase relationships between the SNP alleles and the causative alleles will serve to destroy evidence of associations. Here, inconsistent phase means that the same SNP shows LD to the QTL but a different allele at the SNP shows a positive relationship to the QTL while consistent phase means that the same allele at the SNP has a positive relationship to the QTL. Obviously, combining data from SNPs with consistent phases in different breeds will reinforce the association. However, recent attempts to weight a genomic relationship matrix (GRM) using either LD phase consistency between breeds or estimates of marker effects have failed to improve the accuracy of genomic predictions [8]. It should be noted that while consistency of phase of allele effects is aligned with a positive genetic correlation between phenotypes of the two breeds and might be seen as a proxy for positive genetic correlation, it is not equivalent to a positive genetic correlation.

Instead of merely considering phase or allele effect consistency to improve the accuracy of genomic predictions, one could directly use genetic correlations between breeds for the same phenotype. When pedigree information allows common relatives of two populations to be traced, the genetic correlation for the same phenotype across the two populations can be estimated, e.g. [15, 16]. The larger is the genetic correlation, the larger is the benefit from combining populations for genomic prediction. Moreover, when the pedigree is not deep enough, relatedness among individuals can be inferred using SNP genotype similarity, and the genetic correlation can then be estimated by using the GRM.

To determine whether it is feasible to use genomic correlations to improve genetic predictions, we used data on 1829 Brahman (BB) and 1973 Tropical Composite (TC) cows and bulls genotyped for 71,726 SNPs that were highly polymorphic in *Bos indicus* cattle. The same phenotype in the two breeds was analysed by fitting a bi-variate model that used both the numerator relationship matrix (NRM) based on pedigree and the GRM. The model allows for estimation of (1) the genomic correlation of a given trait across the two breeds; and (2) the “missing heritability” based on the fraction of genetic variance that is not captured by the markers. We showed that building a GRM by using a carefully selected set of SNPs, either with the same or different LD phases between SNP alleles and the QTL, and with SNP effects on the extremes of the distribution, resulted in partial recovery of the missing heritability. It also affected the estimates of genomic correlations, which in turn affected the accuracy of the genomic predictions.

## Methods

### Animals, phenotypes and genotypes

Animal Care and Use Committee approval was not obtained for this study because no new animals were handled in this experiment. The experiment was performed on trait records and DNA samples that had been collected previously. The resource population used in this study was established by the Cooperative Research Centre for Beef Genetic Technologies (Beef CRC) to understand the genetic links between adaptability and components of herd profitability in northern Australia [17].

Animals, phenotypes and genotypes used in this study were a subset of those used in Porto-Neto et al. [18]. In brief, we used data on 1829 Brahman (BB) and 1973 Tropical Composite (TC) cows and bulls genotyped using either the BovineSNP50 [19] or the BovineHD (Illumina Inc., San Diego, CA) that includes more than 770,000 SNPs. Animals that were genotyped with the lower density array had their genotypes imputed to higher density based on the genotypes of relatives, as described previously [11]. The imputation was performed within-breed based on 30 iterations of BEAGLE [20], using 519 Brahman and 351 Tropical Composite animals genotyped with the BovineHD as reference. From the resulting 729,068 SNP genotypes per individual, we extracted the genotypes from 71,726 SNPs that were highly polymorphic in *B. indicus* cattle (GGP Indicus HD Chip; http://www.neogeneurope.com/Agrigenomics/pdf/Slicks/NE_GeneSeekCustomChipFlyer.pdf).

We chose five phenotypes of relevance to tropical adaptation: (1) NAVEL: penile sheath score expressed as the correlated trait navel score in females and scored from 1 (very pendulous) to 9 (extremely tight against the ventral surface of the animal); (2) COLOR: coat colour scored on a light (1) to dark (6) scale; (3) COAT: coat score subjectively scored from an extremely short and slick coat (1) to a very woolly coat (7), which was subsequently converted to a continuous 21 points scale); (4) COND: body condition score (1–5, which was subsequently converted to a continuous 15 points scale); and (5) YWT: yearling weight (kg).

**Fig. 1:**
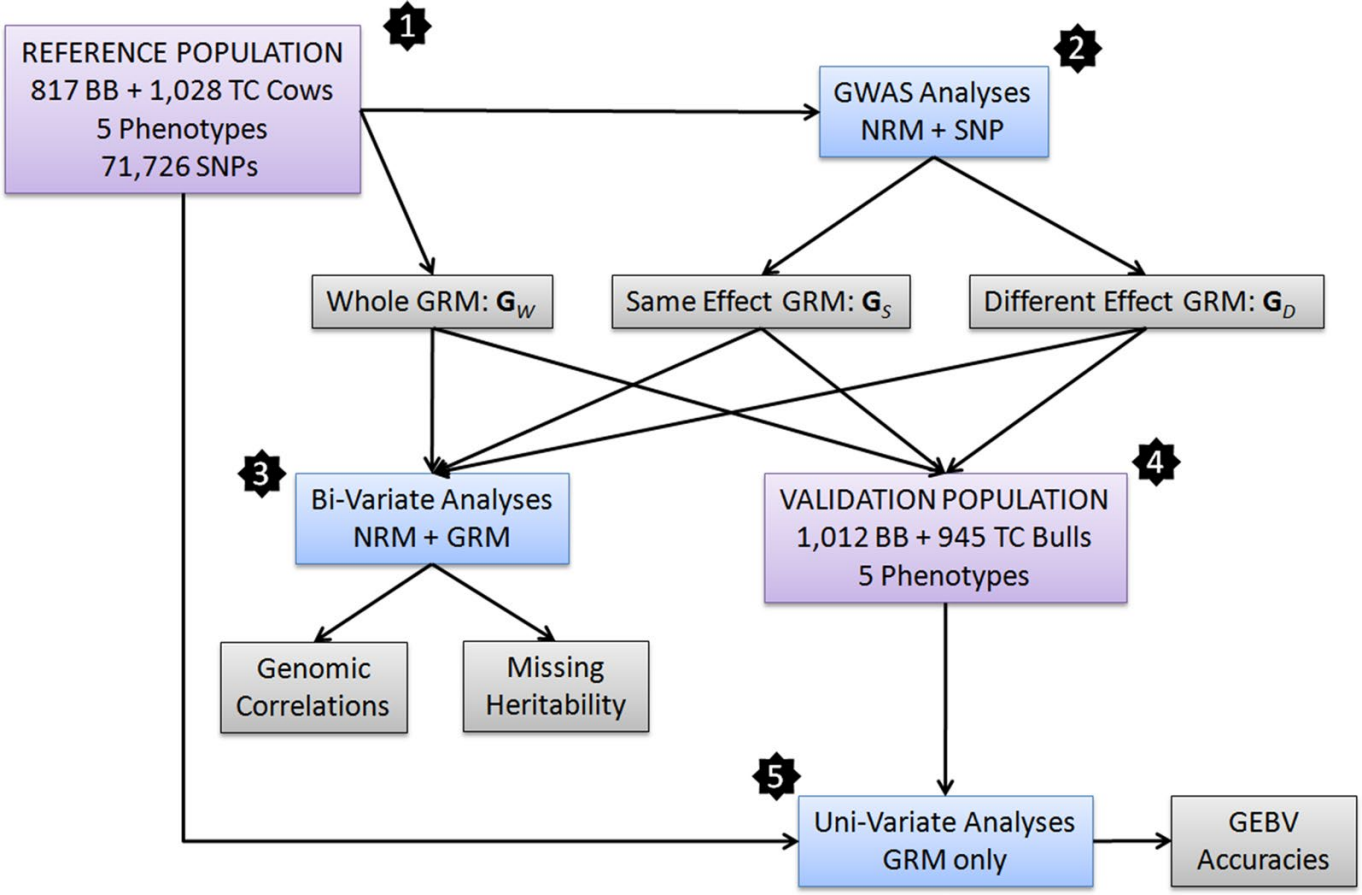
Flowchart of the analyses. *1* A reference population was created by merging data from 817 Brahman (BB) and 1028 Tropical Composite (TC) cows with measures on five traits and genotypes on 71,726 SNPs. A genomic relationship matrix (GRM) was constructed using the whole set of SNPs and termed **G**_*W*_. *2* GWAS analyses were performed separately for each breed using the pedigree-based numerator relationship matrix (NRM). Based on these GWAS, we compiled the set of SNPs with estimated effects in the same and opposite directions in the two breeds, the corresponding GRM were named **G**_*S*_ and **G**_*D*_. *3* The three GRM were compared for their ability to produce estimates of genomic correlation and fractions of missing heritability using a series of bi-variate analyses, where the same phenotype was treated as a different trait in each breed and contained two additive genetic components, NRM and GRM. *4* A validation population was assembled by merging data from 1012 BB and 945 TC bulls with measures on the same five traits as for the cows in the calibration population. *5* The two populations, calibration and validation, were merged and after treating the phenotypes on the validation individuals as missing values, a series of uni-variate analyses (one for each trait) was undertaken with the GRM, either from **G**_*W*_, **G**_*S*_ or **G**_*D*_ as the only additive genetic component. Based on these analyses, the accuracies of the genomic predictions were computed and compared

Figure 1 provides an overview of the analyses undertaken in this study. The available two-breed dataset was partitioned into a reference (or calibration) and a validation population based on sex. The reference population was composed of cows, while the validation population was composed of bulls. It is important to note that the bulls in the validation population were the progeny of cows in the calibration population. The number of sires and mother-son pairs in each calibration/validation population was as follows: (1) for the Brahman population, the 817 calibration cows were from 55 sires and the 1012 validation bulls were also from 55 sires, which were all different from the 55 sires of the validation cows; moreover, 665 cows had sons in the validation population, averaging 1.54 sons per cow and ranging from one (for 382 cows) to four (for 14 cows); (2) for the Tropical Composite population, the 1028 calibration cows were from 52 sires and the 945 validation bulls were from 56 sires, with only one common sire (siring three cows and 27 bulls); moreover, 673 cows had sons in the validation population, averaging 1.44 sons per cow and ranging from one (for 405 cows) to three (for 30 cows).

### Pedigree-based within-breed estimates of heritability and genetic correlations

For each breed, a 10-variate analysis was performed, using the five traits separately for cows and bulls. The mixed-model that we selected had already been used on this population [18] and included the fixed effects of contemporary group (combination of herd of origin, year of birth and grow-out location), age of dam and percent of indicine ancestry of the individual as a covariate. The model for yearling weight contained yearling age as an additional fixed covariate. Additive polygenic random effects were fitted using the pedigree-based relationship matrix.

REML estimates for variance components, heritabilities and genetic correlations for these within-breed analyses were obtained using VCE 6.0.2 (ftp://tzv.fal.de/pub/vce6/) because it provides standard errors for parameter estimates and best linear unbiased estimates (BLUE) solutions to the fixed effects. These BLUE are used to compute adjusted phenotypes which will be needed in the computation of accuracy and bias of genomic estimated breeding values (GEBV).

### REML estimation of genetic parameters and fraction of missing heritability

Each phenotype was treated as a different trait in each breed and analysed using the following bi-variate linear mixed model:

$$\begin{aligned} \left[ {\begin{array}{*{20}c} {{\mathbf{y}}_{\rm{BB}} } \\ {{\mathbf{y}}_{\rm{TC}} } \\ \end{array} } \right] &= \left[ {\begin{array}{*{20}c} {{\mathbf{X}}_{\rm{BB}} } & {\mathbf{0}} \\ {\mathbf{0}} & {{\mathbf{X}}_{\rm{TC}} } \\ \end{array} } \right]\,\left[ {\begin{array}{*{20}c} {{\varvec{\upbeta}}_{\rm{BB}} } \\ {{\varvec{\upbeta}}_{\rm{TC}} } \\ \end{array} } \right]\, \hfill \\ &\quad + \left[ {\begin{array}{*{20}c} {{\mathbf{Z}}_{\rm{BB}} } & {\mathbf{0}} \\ {\mathbf{0}} & {{\mathbf{Z}}_{\rm{TC}} } \\ \end{array} } \right]\,\left[ {\begin{array}{*{20}c} {{\mathbf{a}}_{\rm{BB}} } \\ {{\mathbf{a}}_{\rm{TC}} } \\ \end{array} } \right]\,\, \hfill \\ & \quad+ \left[ {\begin{array}{*{20}c} {{\mathbf{M}}_{\rm{BB}} } & {\mathbf{0}} \\ {\mathbf{0}} & {{\mathbf{M}}_{\rm{TC}} } \\ \end{array} } \right]\,\left[ {\begin{array}{*{20}c} {{\mathbf{u}}_{\rm{BB}} } \\ {{\mathbf{u}}_{\rm{TC}} } \\ \end{array} } \right]\, \hfill \\ & \quad + \left[ {\begin{array}{*{20}c} {{\mathbf{e}}_{\rm{BB}} } \\ {{\mathbf{e}}_{\rm{TC}} } \\ \end{array} } \right] \hfill \\ \end{aligned}$$

where ${\mathbf{y}}_{\rm{BB}}$ and ${\mathbf{y}}_{\rm{TC}}$ are the vectors of the phenotypes of cows from BB and TC breeds, respectively; ${\mathbf{X}}_{\rm{BB}}$ and ${\mathbf{X}}_{\rm{TC}}$ are incidence matrices relating ${\mathbf{y}}_{\rm{BB}}$ and ${\mathbf{y}}_{\rm{TC}}$ with fixed effects in ${\varvec{\upbeta}}_{\rm{BB}}$ and ${\varvec{\upbeta}}_{\rm{TC}}$ (as defined earlier for within-breed analyses); ${\mathbf{Z}}_{\rm{BB}}$ and ${\mathbf{Z}}_{\rm{TC}}$ are incidence matrices relating ${\mathbf{y}}_{\rm{BB}}$ and ${\mathbf{y}}_{\rm{TC}}$ with the random additive genetic animal effects in ${\mathbf{a}}_{\rm{BB}}$ and ${\mathbf{a}}_{\rm{TC}}$ using the pedigree-based relationship matrix; ${\mathbf{M}}_{\rm{BB}}$ and ${\mathbf{M}}_{\rm{TC}}$ are incidence matrices relating ${\mathbf{y}}_{\rm{BB}}$ and ${\mathbf{y}}_{\rm{TC}}$ with the random additive genetic animal effects in ${\mathbf{u}}_{\rm{BB}}$ and ${\mathbf{u}}_{\rm{TC}}$ using the marker-based relationship matrix; and ${\mathbf{e}}_{\rm{BB}}$ and ${\mathbf{e}}_{\rm{TC}}$ are vectors of random residual effects associated with measurements in ${\mathbf{y}}_{\rm{BB}}$ and ${\mathbf{y}}_{\rm{TC}}$, respectively.Note that the dimensions of ${\mathbf{y}}_{\rm{BB}}$ and ${\mathbf{y}}_{\rm{TC}}$ are equal to the number of BB and TC records, respectively. However, ${\mathbf{a}}_{\rm{BB}}$ and ${\mathbf{a}}_{\rm{TC}}$ have the same dimension and are equal to the number of BB and TC animals. These two populations are not related through the pedigree. Therefore, solutions for TC animals in ${\mathbf{a}}_{\rm{BB}}$ (as well as the solutions for BB animals in ${\mathbf{a}}_{\rm{TC}}$) are zero (in fact, they are not estimable).

Random effects were assumed to follow a normal distribution with zero mean and a variance–covariance matrix as follows:

$$V\left[ {\begin{array}{*{20}c} {{\mathbf{a}}_{\rm{BB}} } \\ {{\mathbf{a}}_{\rm{TC}} } \\ {{\mathbf{u}}_{\rm{BB}} } \\ {{\mathbf{u}}_{\rm{TC}} } \\ {{\mathbf{e}}_{\rm{BB}} } \\ {{\mathbf{e}}_{\rm{TC}} } \\ \end{array} } \right]\; = \,\,\left[ {\begin{array}{*{20}c} {\sigma_{{{\text{a}}_{\rm{BB}} }}^{ 2} {\mathbf{A}}} & {\mathbf{0}} & {\mathbf{0}} & {\mathbf{0}} & {\mathbf{0}} & {\mathbf{0}} \\ {\mathbf{0}} & {\sigma_{{{\text{a}}_{\rm{TC}} }}^{ 2} {\mathbf{A}}} & {\mathbf{0}} & {\mathbf{0}} & {\mathbf{0}} & {\mathbf{0}} \\ {\mathbf{0}} & {\mathbf{0}} & {\sigma_{{{\text{u}}_{\rm{BB}} }}^{ 2} {\mathbf{G}}} & {\sigma_{{{\text{u}}_{\rm{BB,TC}} }} {\mathbf{G}}} & {\mathbf{0}} & {\mathbf{0}} \\ {\mathbf{0}} & {\mathbf{0}} & {\sigma_{{{\text{u}}_{\rm{BB,TC}} }} {\mathbf{G}}} & {\sigma_{{{\text{u}}_{\rm{TC}} }}^{ 2} {\mathbf{G}}} & {\mathbf{0}} & {\mathbf{0}} \\ {\mathbf{0}} & {\mathbf{0}} & {\mathbf{0}} & {\mathbf{0}} & {\sigma_{{{\text{e}}_{\rm{BB}} }}^{ 2} {\mathbf{I}}} & {\mathbf{0}} \\ {\mathbf{0}} & {\mathbf{0}} & {\mathbf{0}} & {\mathbf{0}} & {\mathbf{0}} & {\sigma_{{{\text{e}}_{\rm{TC}} }}^{ 2} {\mathbf{I}}} \\ \end{array} } \right]\;,$$

where $\sigma_{{{\text{a}}_{\rm{BB}} }}^{ 2}$ and $\sigma_{{{\text{a}}_{\rm{TC}} }}^{ 2}$ are the additive genetic variances due to pedigree-based relationships in the BB and TC populations (known to be zero between breeds since they have no common ancestors in the pedigree), respectively; $\sigma_{{{\text{u}}_{\rm{BB}} }}^{ 2}$ and $\sigma_{{{\text{u}}_{\rm{TC}} }}^{ 2}$ are the additive genetic variances due to marker-based relationships within the BB and TC breeds, respectively; $\sigma_{{{\text{u}}_{\rm{BB,TC}} }}$ is the additive genetic covariance due to marker-based relationships between the BB and the TC populations; $\sigma_{{{\text{e}}_{\rm{BB}} }}^{ 2}$ and $\sigma_{{{\text{e}}_{\rm{TC}} }}^{ 2}$ are the residual variances associated with measurements in BB and TC, respectively; **A** is the pedigree-based numerator relationship matrix [21], which was computed recursively based on three generations of ancestors; and **G** is the GRM relating animals of the same and different breeds and generated from the 71,726 SNP genotypes according to the method proposed by VanRaden [22], with the modification of Karoui et al. [23] to make it invertible:

$${\mathbf{G}}\; = \; 0. 9 5\,\times \frac{{{\mathbf{SS}}^{T} }}{{2\sum {p_{i} (1 - p_{i} )} }}\, + \,0.05\,\times \,{\mathbf{I}},$$

where **S** is the centered matrix relating SNP genotypes (coded 0, 1 or 2) in columns with animals in rows, and $p_{i}$ is the frequency of the second allele of the *i*-th SNP in the across-breed dataset, and **I** is an identity matrix included to make **G** invertible by enlarging the diagonal elements.

It is important to note that, although both relationship matrices, **A** and **G**, were built across both breeds (BB and TC), the off-diagonal elements in **A** relating BB with TC animals were zero as they were unlinked via pedigree. However, off-diagonal elements in **G** relating BB with TC animals were not necessarily zero since they could still be related through allele sharing at the genotyped SNPs. This non-zero relationship between animals across populations made it possible to estimate $\sigma_{{{\text{u}}_{\rm{BB,TC}} }}$, which in turn permitted the computation of the genomic correlation between the given phenotype in the two breeds as follows:

$$r_{\text{G}} \; = \;\frac{{\sigma_{{{\text{u}}_{\rm{BB,TC}} }} }}{{\sqrt {\sigma_{{{\text{u}}_{\rm{BB}} }}^{ 2} \times \sigma_{{{\text{u}}_{\rm{TC}} }}^{ 2} } }}.$$

Variance components were estimated via REML using the Qxpak5 software program [24].

Following Román-Ponce et al. [25], estimates of missing heritability were obtained from the fraction of genetic variance not captured by the SNPs used to generate **G** and was estimated as follows:

$$C_{miss}^{\rm{BB}} \; = \;1 - \,\frac{{\sigma_{{{\text{u}}_{\rm{BB}} }}^{ 2} }}{{\sigma_{{{\text{u}}_{\rm{BB}} }}^{ 2} + \sigma_{{{\text{a}}_{\rm{BB}} }}^{ 2} }}.$$

The same method was applied for estimating $C_{miss}^{\rm{TC}}$ for the fraction of genetic variance not captured by the SNPs in the TC population.

### GWAS and selection of SNPs

In addition to the bi-variate analyses, we performed genome-wide association analyses (GWAS) separately within each population of cows (BB and TC) and for each of the five traits using Qxpak5 software [24]. The GWAS were performed with REML using a uni-variate mixed model which included the following components: (1) the fixed effects as defined for the bi-variate models; (2) the random additive genetic effect in ${\mathbf{a}}_{\rm{BB}}$ (or ${\mathbf{a}}_{\rm{TC}}$ in the analysis of the TC population) using the pedigree-based relationship matrix; (3) the SNP genotype (coded 0, 1 or 2) as an additional fixed linear covariate; and (4) the random residual.

The SNP effect estimates for a given trait in the population of BB cows were compared with those for the same trait in the population of TC cows to select SNPs that had large effects in both populations and in either the same or opposite directions. For each trait, the 7173 (or 10 %) most significant SNPs and in the same direction in both breeds were selected. The resulting five lists (one for each trait) were merged into a single list namely “list-of-same”. Similarly, the five lists of the 10 % most significant SNPs with estimated effects in opposite directions in the two breeds were merged into a single list, namely “list-of-different”. Finally, all overlapping SNPs between “list-of-same” and “list-of-different” were removed.

This selection approach resulted in two lists of non-overlapping SNPs (16,207 and 16,951 in “list-of-same” and “list-of-different”, respectively). The percentage of SNPs that influenced one, two, three, four or all five traits was 75.2, 19.7, 4.2, 0.89 and 0.08 % for the 16,207 SNPs in “list-of-same”; and 76.9, 19.3, 3.4, 0.38 and 0.05 % for the 16,951 SNPs in “list-of-different”.

These two lists were used to construct: **G**_*S*_ i.e. the GRM built using the SNPs in “list-of-same”; and **G**_*D*_ i.e. the GRM built using the SNPs in the “list-of-different”. The performance of **G**_*S*_ and **G**_*D*_ was explored in a new set of bi-variate mixed models, which also included the polygenic effect of the pedigree-based numerator relationship matrix **A**. The resulting estimates of variance components and the fraction of missing heritability were compared to those obtained with **G**_*W*_, the GRM built using the entire list of 71,726 SNPs.

### Genomic prediction models

For the genomic prediction models, we merged data from both breeds and both sexes into a single dataset, and carried out REML analyses using a uni-variate mixed-effect model that used the random additive genetic animal effects based on the marker-based relationship matrix as follows:

$${\mathbf{y}}\; = \;{\mathbf{X}}{{\varvec\upbeta }}\, + \,{\mathbf{Mu}}\, + {\mathbf{e}},$$

where ${\mathbf{y}}$ is the vector of phenotypes across both breeds and sexes and the remaining effects are defined as before, except that ${\mathbf{u}}\;\sim \;N({\mathbf{0}},{\mathbf{G}}\sigma_{{\mathbf{u}}}^{2} )$, and matrix **G** was based on one of three possible options:

1. **G**_*W*_ = the GRM built with the whole set of 71,726 SNP genotypes;
2. **G**_*S*_ = the GRM built with the set of SNPs that had estimated effects in the same direction in the two breeds and across all five traits;
3. **G**_*D*_ = the GRM built with the set of SNPs that had estimated effects in opposite directions in the two breeds and across all five phenotypes.

### Accuracy and bias of genomic predictions

In the above-mentioned models, the phenotypes from bulls from the two breeds were always treated as missing values. The phenotypes of cows were treated as missing values separately for each breed depending on the validation population being tested (treated as missing). Therefore, for each phenotype, we explored two genomic prediction models: (1) one with BB cows as the only observed phenotype; and (2) one with TC cows as the only observed phenotype.

This scheme allowed us to investigate the accuracy of genomic predictions using three approaches:

1. Using cows of one breed to predict cows of the other breed.
2. Using cows of one breed to predict bulls of the same breed.
3. Using cows of one breed to predict bulls of the other breed.

While the first approach does not represent an independent validation analysis because the cows from both breeds were used in the identification of SNPs for **G**_*S*_ and **G**_*D*_, it establishes the upper bounds of the achievable accuracies. Prediction accuracy was estimated from the correlation between the genomic estimated breeding value (GEBV) and the observed phenotype of the validation animals adjusted for the fixed effects estimated from the within-breed 10-variate analyses (i.e., using the five traits separately for cows and bulls). Prediction bias was assessed from the regression coefficient of adjusted phenotypes on GEBV.

## Results

**Fig. 2:**
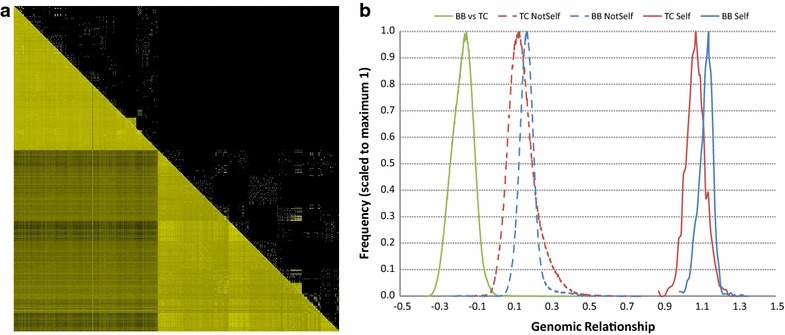
Genomic relationships. **a** Heat map of the relationship matrices. Above the diagonal is the pedigree-based numerator relationship matrix; below the *diagonal* is the SNP-based genomic relationship matrix. Animals were sorted such that the 817 Brahman cows appear first, followed by the 1028 Tropical Composite cows. The *colour scheme* goes from black to *yellow* for relationships of zero (none) and one (self), respectively. **b** Distribution of the genomic relationship coefficients within and across breeds

Table 1 provides summary statistics including number of records, means and standard deviations for the five traits included in this study. The objective of splitting the reference and validation populations based on sex was to have both breeds represented in each population without any overlapping individuals. It should be noted that, within a breed, bulls in the validation set were progeny of cows in the calibration set. Also, there were no known genetic links at the level of the great-grandparents between the BB and TC individuals, although some animals in the BB and TC samples should clearly have common ancestors because of the use of Brahman animals when the Tropical Composite population was formed [18]. To visualise these links, Fig. 2a provides a heat map of the relationship matrices for cows across the two breeds. The upper diagonal part corresponds to the pedigree-based NRM, while the lower diagonal part corresponds to the marker-based GRM. Some mirror image symmetries are clearly apparent within-breed between the two relationship matrices. However, the main feature of Fig. 2a is the sparsity of the NRM and complete absence of relationships in the block corresponding to BB by TC relationships. In contrast, there were obvious links between the BB and TC individuals based on the GRM. To further support these results, Fig. 2b shows the distributions of genomic relationships within and between breeds. The level of relationships was greater between BB animals than between TC animals. This may be due to the TC sample being larger than the BB sample and the allele frequencies that were computed across breeds. A similar observation was reported by Karoui et al. [23], who compared three dairy cattle breeds and found that the most abundant breed (Holstein) had the lowest level of genomic relationships.

**Table 1:** Means (standard deviations) for the five traits across the calibration and validation populations and the two breeds, Brahman (BB) and Tropical Composite (TC)

Trait	Calibration (cows)		Validation (bulls)	
	BB N = 817	TC N = 1028	BB N = 1012	TC N = 945
Navel score	5.40 (1.04)	7.90 (1.04)	4.19 (0.93)	6.86 (1.54)
Coat score	4.92 (1.17)	7.38 (2.32)	5.14 (1.62)	5.45 (1.68)
Coat colour	3.38 (0.64)	3.75 (0.82)	3.17 (1.01)	3.89 (0.89)
Body condition	8.04 (0.86)	7.32 (0.82)	6.71 (0.50)	6.58 (0.54)
Yearling weight	209.87 (29.83)	217.07 (27.32)	243.79 (30.72)	284.09 (34.53)

*N* number of individuals

For within-breed analyses, and using the pedigree as the only information to relate animals, Table 2 provides heritability estimates separately for each sex and the genetic correlation for the same trait between the two sexes. All five traits were moderately heritable, with estimates greater than 50 % for 15 out of 20 estimates. Also, estimates of genetic correlations were high and close to 1 for most traits, with the possible exception of YWT for the BB animals (0.711 ± 0.068) and COND for the TC animals (0.621 ± 0.066). Deviations from 1 of the estimates of genetic correlation anticipate a potential difficulty of using cows (parents in this case) to predict bulls (progeny in this case).

**Table 2:** Within-breed pedigree-based heritability (h^2^) estimates (standard errors) for the five traits across the two sexes (cows and bulls) and breeds, Brahman (BB) and Tropical Composite (TC), and genetic correlations (r_g_) for the same trait in two sexes

Trait	BB			TC		
	h_cows_^2^	h_bulls_^2^	r_g_	h_cows_^2^	h_bulls_^2^	r_g_
Navel score	0.52 (0.05)	0.53 (0.06)	0.94 (0.04)	0.57 (0.05)	0.72 (0.06)	0.88 (0.02)
Coat score	0.66 (0.05)	0.32 (0.06)	0.81 (0.09)	0.54 (0.04)	0.62 (0.05)	0.88 (0.04)
Coat colour	0.57 (0.04)	0.59 (0.05)	0.95 (0.02)	0.57 (0.05)	0.52 (0.04)	0.94 (0.03)
Body condition	0.56 (0.05)	0.46 (0.06)	0.83 (0.05)	0.47 (0.04)	0.41 (0.05)	0.62 (0.07)
Yearling weight	0.45 (0.06)	0.61 (0.07)	0.71 (0.07)	0.55 (0.05)	0.68 (0.05)	0.96 (0.02)

Table 3 lists the results from the bi-variate analyses of the five traits when treating the same trait measurement as a different phenotype in each population. The percentage of genetic variance explained by the entire set of 71,726 SNPs ranged from 37.5 % for NAVEL for BB cows to 57.6 % for COND for TC cows. Similarly, the percentage of genetic variance not captured by SNPs, i.e. the missing heritability, ranged from 24.0 % for COND for TC cows to 47.8 % for YWT for BB cows. The average missing heritability was 43.3 and 39.4 % for the BB and TC cows, respectively. These values are somewhat larger than the 36.6 % reported by Roman-Ponce et al. [25] across 11 traits, or the 38.5 % reported by Haile-Mariam et al. [26] across 29 traits. Both studies used dairy cattle datasets.

**Table 3:** Estimates of genetic parameters from bi-variate analyses using the GRM built with the whole set of 71,726 SNPs in the two breeds

Trait	BB			TC			*r*_*G*_
	$h_{P}^{2}$	$h_{G}^{2}$	***C***_***miss***_	$h_{P}^{2}$	$h_{G}^{2}$	***C***_***miss***_	
Navel score	0.34	0.38	0.48	0.29	0.50	0.37	0.12
Coat score	0.36	0.39	0.48	0.32	0.44	0.42	0.07
Coat colour	0.33	0.38	0.47	0.34	0.39	0.46	0.07
Body condition	0.21	0.57	0.27	0.18	0.58	0.24	0.63
Yearling weight	0.39	0.42	0.48	0.36	0.39	0.48	0.04

Proportion of variance explained by either pedigree-based ($h_{P}^{2}$) or genomic-based ($h_{G}^{2}$) genetic variance, proportion of missing heritability (*C*_*miss*_) and estimate of genomic correlation (*r*_*G*_) between Brahman (BB) and Tropical Composite (TC) cows for the five traits

With the exception of COND for which the estimated genomic correlation was moderate and equal to 0.63, estimated genomic correlations for all other traits were near 0 but positive. In a study of five traits in dairy cattle, Zhou et al. [8] reported estimated genomic correlations that ranged from 0.37 to 0.58. Also in dairy cattle breeds, Karoui et al. [23] reported genetic (genomic) correlation estimates that ranged from −0.01 for fertility between Montbéliarde and Normande cattle to 0.79 for milk production between Montbéliarde and Holstein cattle. In our study, the results for four of the traits suggest that merging the two datasets would not greatly enhance the prediction accuracies of genomic selection.

SNPs that are not associated to a trait may add errors to a prediction so we repeated the previous analyses by restricting the SNPs used to construct genomic relationships to those that had consistent phases (defined above) in both breeds. Note that SNPs for all five traits were included in the pool of SNPs, although a SNP did not usually show significant effects for all five traits. The same set of parameter estimates was explored using the GRM built with those SNPs (**G**_*S*_; Table 4), as well as using the GRM built with the SNPs with inconsistent effects in both breeds (**G**_*D*_; Table 5). This resulted in a decrease in the variance explained by the NRM and a large increase in the proportion of genetic variance captured by SNPs, which resulted in a much lower fraction of missing heritability, which was never greater than 17.5 %.

**Table 4:** Bi-variate analyses using **G**_*S*_ (i.e. the GRM built with the SNPs with effects in the same direction in both breeds)

Phenotype	BB			TC			*r*_*G*_
	$h_{P}^{2}$	$h_{G}^{2}$	***C***_***miss***_	$h_{P}^{2}$	$h_{G}^{2}$	***C***_***miss***_	
Navel score	0.08	0.88	0.08	0.09	0.86	0.10	0.93
Coat score	0.16	0.77	0.18	0.14	0.79	0.15	0.83
Coat colour	0.09	0.84	0.10	0.06	0.91	0.06	0.92
Body condition	0.10	0.85	0.10	0.08	0.88	0.08	0.92
Yearling weight	0.06	0.92	0.06	0.08	0.88	0.08	0.96

Proportion of variance explained by either pedigree-based ($h_{P}^{2}$) or genomic-based ($h_{G}^{2}$) genetic variance, proportion of missing heritability (*C*_*miss*_) and estimate of genomic correlation (*r*_*G*_) between Brahman (BB) and Tropical Composite (TC) cows for the five phenotypes

**Table 5:** Bi-variate analyses using **G**_*D*_ (i.e. the GRM built with the SNPs with effects in different directions in both breeds)

Phenotype	BB			TC			*r*_*G*_
	$h_{P}^{2}$	$h_{G}^{2}$	***C***_***miss***_	$h_{P}^{2}$	$h_{G}^{2}$	***C***_***miss***_	
Navel score	0.10	0.85	0.11	0.07	0.89	0.08	−0.91
Coat score	0.12	0.83	0.13	0.11	0.83	0.12	−0.89
Coat colour	0.08	0.87	0.08	0.06	0.91	0.06	−0.93
Body condition	0.08	0.88	0.08	0.10	0.84	0.10	−0.95
Yearling weight	0.07	0.90	0.07	0.08	0.88	0.08	−0.95

Proportion of variance explained by either pedigree-based ($h_{P}^{2}$) or genomic-based ($h_{G}^{2}$) genetic variance, proportion of missing heritability (Cmiss) and estimate of genomic correlation (rG) between Brahman (BB) and Tropical Composite (TC) cows for the five phenotypes

Using simulated data, de los Campos et al. [27] showed that using genotypes at QTL when constructing the GRM removes the missing heritability. Conversely, these authors also showed that adding SNPs in imperfect LD with the QTL added considerable uncertainty to the heritability estimates. Using **G**_*S*_ or **G**_*D*_ drastically affected estimates of genomic correlations, with no estimates below 0.83 when using **G**_*S*_ (Table 4) and none above −0.88 when using **G**_*D*_ (Table 5). However, these massive changes are not entirely surprising and can be attributed to bias due to the process used to select SNPs since they have been prioritized using the same data previously used to define **G**_*S*_ and **G**_*D*_. Nevertheless, we conclude that estimates of genomic correlations can be manipulated to a near +1.0 and a near −1.0 when using **G**_*S*_ and **G**_*D*_, respectively.

Selection of the SNPs used in the construction of the GRM also resulted in substantial changes in the accuracy and bias of genomic predictions. While accuracy affects the ranking of individuals, bias affects the range of estimated breeding values. Tables 6, 7, and 8 provide accuracies and biases of genomic predictions across all three possible calibration–validation possibilities, i.e., (1) using cows of one breed to predict cows of the other breed (Table 6), (2) using cows of one breed to predict bulls of the same breed (Table 7), and (3) using cows of one breed to predict bulls of the other breed (Table 8). Again, the large impact on the accuracies of genome predictions presented in Table 6 can be attributed to bias in selecting SNPs. This bias was particularly pronounced when using **G**_*S*_ and BB cows to select TC cows (average bias = 2.123; Table 6). Nevertheless, these results are interesting since they demonstrate the extent to which the accuracy and bias of genomic predictions can be affected by selecting the SNPs that are used to build the GRM.

**Table 6:** Accuracy and bias of genomic predictions using cows of one breed to predict cows of the other breed and the relationship matrix based on the whole set of 71,726 SNPs (**G**_*W*_) or the SNPs with effect estimates with the same sign in the two breeds (**G**_*S*_) or the SNPs with effect estimates with different signs in the two breeds (**G**_*D*_)

Trait	BB cows: TC cows			TC cows: BB cows		
	**G**_*W*_	**G**_*S*_	**G**_*D*_	**G**_*W*_	**G**_*S*_	**G**_*D*_
Accuracy						
Navel score	0.40	0.70	−0.47	0.14	0.58	−0.60
Coat score	0.11	0.64	−0.54	0.07	0.53	−0.57
Coat colour	0.05	0.65	−0.62	0.13	0.67	−0.66
Body condition	0.20	0.55	−0.48	0.20	0.65	−0.57
Yearling weight	0.07	0.45	−0.43	0.09	0.43	−0.38
Average	0.17	0.60	−0.51	0.13	0.57	−0.56
Bias						
Navel score	1.65	1.48	−2.00	0.61	1.12	−2.03
Coat score	0.92	3.77	−3.16	0.28	0.76	−1.01
Coat colour	0.42	2.29	−2.49	0.83	1.24	−1.38
Body condition	1.42	1.46	−1.85	1.22	2.04	−2.27
Yearling weight	0.57	1.61	−1.72	0.73	1.56	−1.70
Average	1.00	2.12	−2.24	0.73	1.35	−1.68

**Table 7:** Accuracy and bias of genomic selection using cows of one breed to predict bulls of the same breed and the relationship matrix using either the whole set of 71,726 SNPs (**G**_*W*_) or the SNPs with effects in the same directions in both breeds (**G**_*S*_) or the SNPs with effects in different directions in both breeds (**G**_*D*_)

Phenotype	BB cows:BB bulls			TC cows:TC bulls		
	**G**_*W*_	**G**_*S*_	**G**_*D*_	**G**_*W*_	**G**_*S*_	**G**_*D*_
Accuracy						
Navel score	0.23	0.25	0.21	0.56	0.56	0.33
Coat score	0.04	0.05	0.05	0.29	0.29	0.28
Coat colour	0.40	0.40	0.37	0.31	0.30	0.28
Body condition	0.15	0.17	0.11	0.11	0.16	0.06
Yearling weight	0.20	0.20	0.17	0.20	0.23	0.18
Average	0.21	0.21	0.18	0.30	0.31	0.22
Bias						
Navel score	0.78	0.69	0.62	1.26	1.07	1.45
Coat score	0.78	0.66	0.64	0.65	0.56	0.61
Coat colour	1.65	1.40	1.42	1.17	0.98	0.96
Body condition	0.39	0.32	0.30	0.40	0.39	0.31
Yearling weight	0.90	0.67	0.60	1.21	1.14	0.99
Average	0.90	0.75	0.72	0.94	0.83	0.87

**Table 8:** Accuracy and bias of genomic selection using cows of one breed to predict bulls of the other breed and the relationship matrix using either the whole set of 71,726 SNPs (**G**_*W*_) or the SNPs with effects in the same directions in both breeds (**G**_*S*_) or the SNPs with effects in different directions in both breeds (**G**_*D*_)

Phenotype	BB cows: TC bulls			TC cows: BB bulls		
	**G**_*W*_	**G**_*S*_	**G**_*D*_	**G**_*W*_	**G**_*S*_	**G**_*D*_
Accuracy						
Navel score	0.32	0.46	−0.09	0.14	0.20	−0.12
Coat score	0.08	0.23	−0.20	0.09	0.05	0.03
Coat colour	−0.06	0.13	−0.17	0.05	0.27	−0.26
Body condition	0.22	0.18	0.07	0.10	0.15	−0.02
Yearling weight	0.09	0.18	−0.04	0.10	0.21	−0.05
Average	0.13	0.24	−0.09	0.10	0.18	−0.08
Bias						
Navel score	1.95	1.93	−0.71	0.43	0.45	−0.49
Coat score	0.82	0.93	−1.25	0.41	0.26	0.10
Coat colour	−0.50	0.84	−1.24	0.46	1.04	−1.15
Body condition	1.04	0.44	−0.07	0.47	0.39	−0.07
Yearling weight	1.32	1.00	−0.25	0.70	0.91	−0.29
Average	1.13	1.03	−0.71	0.49	0.61	−0.45

However, selection of SNPs did not affect either the accuracy or the bias of genomic predictions when these predictions were made within-breed (Table 7). The 16,207 and 16,961 SNPs included in **G**_*S*_ and **G**_*D*_, respectively, were sufficient to allow for a genomic prediction accuracy equivalent to that provided by **G**_*W*_ (the GRM built with the whole set of 71,726 SNPs). Indeed, the accuracies using **G**_*W*_ and **G**_*S*_ were effectively identical, although only 23 % of the SNPs were used to calculate **G**_*S*_. Accuracies using **G**_*D*_ were slightly lower for all traits, except for NAVEL in the TC sample, for which the accuracy was substantially lower. Since a major gene is known to affect NAVEL [18], the “list-of-different” SNPs used in **G**_*D*_ might lack markers in a critical part of the genome so that all the local variance was not captured. In spite of the negative estimates for genomic correlations when using the “list-of-different” SNPs, the accuracies using **G**_*D*_ were all positive when the predictions were made within-breed.

Accuracies of genomic predictions across breeds and sexes (Table 8) showed a marked improvement or decline when using **G**_*S*_ or **G**_*D*_, respectively. Averaged across all traits, using **G**_*S*_, led to a 79 % (from 0.13 to 0.24) increase in prediction accuracy for TC bulls based on BB cows, along with a decrease of 0.10 in bias (from 1.13 to 1.03). Similarly, using **G**_*S*_, led to an average increase in prediction accuracy for BB bulls based on TC cows of 84 % (from 0.10 to 0.18) along with an improvement of 0.12 in bias (from 0.49 to 0.61). However, the use of **G**_*D*_ led to a major decrease in accuracy when selecting bulls of one breed based on cows of another breed as can be seen in the average negative accuracy of 0. Also, negative estimates of bias were obtained when using **G**_*D*_ in across-breed predictions, either within-sex (Table 6) or across sexes (Table 8).

## Discussion

There is still extensive room for improvement in genomic prediction especially when the numbers of sampled animals are small. In this study on the use of genetic correlation to improve genomic prediction we have: (1) shown the hidden relationship between breeds that is uncovered by using SNPs; (2) estimated the genetic correlation between breeds for five traits and the missing heritabilities; (3) found that the genetic correlations were generally low, thus, although we have uncovered the genetic relationships, combining data for the two breeds would not materially add to the improvement in genomic prediction; and (4) shown that the effects of genetic correlations in genomic prediction can be manipulated by the choice of SNPs and can result in both changes to accuracy and increases in bias in prediction across breeds although accuracy and bias of prediction within breeds appears unaffected.

Our results have some limitations but they do go beyond what others have found in the field. For example, the allele frequencies used in the computation of the GRM were estimated from the combined two-breed genotype data. Other choices such as breed-specific allele frequencies or the use of a common base population could be explored e.g., as in [6, 14, 28, 29]. These recently described alternatives for SNP selection take different marker allele frequencies between breeds and crossbred data into account. In addition, the process for selecting SNPs to be included in **G**_*S*_ and **G**_*D*_ was intrinsically simple, i.e. we selected the 10 % SNPs that were most associated with each trait and had the same direction of effects (**G**_*S*_) or opposite directions of effects (**G**_*D*_) and then avoided any SNP that fell in both categories. Other approaches for the selection of SNPs should be investigated. For instance, an approach that focuses on SNPs in coding regions or in DNA regions that are not coding for proteins but are functional could be explored. In this regard, Koufariotis et al. [30] found that regulatory and coding genome regions are enriched for trait-associated variants in dairy and beef cattle. Similarly, Bolormaa et al. [31] reported a higher likelihood of trait association for SNPs that are located in a region 100 kb before and after a gene. Su et al. [32] showed that weighting SNPs by the significance of their association to a trait when constructing the GRM increased the accuracy by 1.7 %. Finally, González-Recio et al. [33] first coined the term “genomic correlation” for the genetic correlation estimated using SNP genotypes. Working with a single breed, Australian Holsteins, these authors explored a series of tri-variate models that included two components of feed efficiency and a third trait relevant to the dairy industry. Their approach differs from that presented here in that a single phenotype is treated as a separate trait in two breeds. Therefore, our estimates of genomic correlation indicate the strength by which the genes affecting one trait in one breed are related with the genes affecting the same trait in another breed. Similar to our approach, Karoui et al. [23] reported estimated genetic correlations between three dairy cattle breeds that were moderately high for milk production and fat content, and relatively low for female fertility.

Our work illustrates some of the challenges ahead to improve genomic prediction. First, in the construction of relationship matrices, the exclusion of phenotypically relevant variants and the inclusion of irrelevant variants both have deleterious effects [34]. Recently, de los Campos et al. [27] presented a formal proof for the expectation that there should be no missing heritability if all causal variants were in the SNP panel. They also implied that by using sequence data there should be no missing heritability. However, Pérez-Enciso et al. [35] predicted that using sequence data was truly beneficial only if accurate biological information was available to assist SNP selection. Their simulations showed that absence of or incomplete inclusion of all causal genes resulted in a rapid decrease in prediction accuracy. Second, Zhou et al. [8] found no improvement in the accuracy of GEBV when weighting the two-breed GRM by LD phase consistencies and marker effects. While the approach that they followed was different to that presented here, the authors attributed their unsuccessful results to the fact that the SNP density in their study was not sufficiently high to preserve the LD between SNPs and QTL across breeds. Instead, our results suggest that marker density in itself will not improve predictions, but if attempts are made to select SNPs that are associated and consistently in the same phase to the trait, these will improve prediction accuracies. Since most of the genetic variance was due to the polygenic background of the traits analysed, we conclude that it may be more important to maximize the genomic correlation between breeds by carefully selecting the SNPs to be used in building the GRM than to preserve specific QTL and LD relationships between breeds.

Some of the areas that will require more research concern highly divergent breeds or sex limited traits. For example, with higher values of the proportion of variation accounted for by the GRM and higher genetic correlations, genomic prediction accuracies improved substantially, especially when comparing cows to cows. It is natural to expect that phenotypes that are measured on one sex are likely to be more representative of animals of that sex, and thus, prediction accuracies for bulls using cows as reference animals are expected to be less accurate. Indeed, while none of the traits explored here were sex-limited, the estimated genetic correlations for some of the traits in the two sexes deviated from 1. Nevertheless, since cows were used as the calibration sample, it is possible that a component of the improved prediction for cow data is due to this fact. We suggest that this approach should be used with caution and tested on other datasets to determine its generality. In addition, when analysing the impact of genetic distance between populations on the accuracy of GEBV, Varona et al. [36] showed that the estimated correlations between estimates of SNP effects moved from near 1 to near 0 as the genetic distance between populations increased. With a decreased correlation between estimates of SNP effects, a model that assumed SNP effects to be different but correlated in the two populations resulted in more accurate predictions than models that assumed either independent SNP effects or identical SNP effects.

Finally, the changes in bias and accuracy when the SNP lists were altered are interesting and instructive. We found that selecting SNPs associated with at least one trait that had the same direction of effect in the two breeds had a major impact on improving the amount of information contained in the GRM. This method substantially reduced both the amount of information explained by the pedigree and the missing heritability, which in our data, appeared to be highly correlated. Adjusting the GRM and NRM to a common base population using the approaches of Roman-Ponce et al. [25] did not significantly affect the estimates of variance components, with the possible exception of NAVEL in the TC sample (results not shown), which we attribute to the low level of inbreeding in our populations. It is important to note that the selected SNPs not only included SNPs that were associated with the trait that was being predicted but also SNPs with effects in the same direction for all traits, even if the association was not statistically significant. This is relevant because it demonstrates that the improvements are not due to a form of bias in selection because the traits are generally uncorrelated to each other. Selecting SNPs of opposite phases resulted in negative genomic correlations, although they were still extremely high, which suggests that the sign of the SNP effects affects the sign of the correlation but not the level of the significance of the association. This finding should be kept in mind when trying to recover information via genomic correlations.

## Conclusions

We showed that the accuracy of across-breed genomic predictions improved when a subset of SNPs with consistent effects across populations is used to generate the predictions, instead of the randomly selected or equally spaced SNPs currently used for genomic selection. These results offer hope for the effective implementation of genomic prediction in situations where there are many breeds, the sample size is small within any single breed and there are many phenotypes in the breeding objective.
